# Viral replication dynamics: Coinfection and superinfection of Vero cells with Zika virus and West Nile virus or Zika virus and Mayaro virus

**DOI:** 10.1371/journal.pone.0354137

**Published:** 2026-07-24

**Authors:** Larissa Krokovsky, Mariana Garrido de Castro, Amanda J. M. Williams, Fiona F. Hunter

**Affiliations:** 1 Department of Biological Sciences, Brock University, St. Catharines, Ontario, Canada; 2 Entomogen Inc., St. Catharines, Ontario, Canada; The University of Queensland, AUSTRALIA

## Abstract

Globalization and climate change have contributed to the emergence and spread of arboviruses, with the possibility of their cocirculation in the same geographic region at the same time. Understanding the effects of concurrent infections within a mosquito vector or human host is therefore an emerging focus in arboviral research. Studies using mosquito- and mammalian-derived cell lines provide a controlled framework for studying viral replication dynamics. In this study, Vero cells were infected with either Zika virus (ZIKV) and West Nile virus (WNV) or ZIKV and Mayaro virus (MAYV). In nature, concurrent infections can occur either simultaneously (i.e., coinfections) or sequentially (i.e., superinfections), and both conditions were studied here. Arbovirus replication was assessed 6 days post-infection using RT-qPCR, along with corresponding cytopathic effects on cells. Single infections were used as comparators to the coinfection and superinfection assays. Coinfections (ZIKV/WNV and ZIKV/MAYV) resulted in viral interference, with ZIKV RNA levels reduced when cells were coinfected with either WNV or MAYV. Viral accommodation was observed when ZIKV was the first to infect the cells during superinfections. In contrast, superinfection exclusion was observed when ZIKV was introduced to cells that were already infected with another virus. RNA production of WNV and MAYV was not affected by the presence of ZIKV in any of the conditions investigated. Understanding replication dynamics in *in vitro* coinfection and superinfection scenarios is the first step toward addressing the knowledge gap concerning cocirculating arboviruses.

## Introduction

Millions of humans are affected by arthropod-borne viruses (arboviruses) each year as these pathogens (re)emerge worldwide [1,2]. These viruses are maintained in nature in cycles involving hematophagous mosquitoes or ticks and vertebrate hosts and can pose a severe threat to public health, with substantial economic burden, morbidity, and mortality worldwide [3]. Most disease-causing arboviruses are transmitted by the bite of an infected mosquito and are distributed in four viral families: *Bunyaviridae*, *Flaviviridae*, *Reoviridae*, and *Togaviridae*. Human disease-causing arboviruses often co-circulate, being found in intersecting geographical regions such as the Americas and tropical areas [4–6].

In South America, the cocirculation of arboviruses such as dengue (DENV), Zika (ZIKV), chikungunya (CHIKV), Oropouche (OROV), and Mayaro (MAYV) caused more than 13 million cases in 2024 [7]. This intense propagation is more evident in some countries, which are considered hotspots for outbreaks and clinical complications. Brazil is considered the most affected country, and in 2024, DENV alone accounted for more than 10 million cases. [8]. Some of these pathogens are considered urban-associated, as mosquitoes such as *Aedes aegypti* and *Culex quinquefasciatus* have adapted to urban environments [2,9,10]. During the 2015–2016 epidemic, ZIKV was associated with a unique clinical pattern in newborns (Congenital Zika Syndrome) and adults (Guillain-Barré Syndrome), resulting in an unprecedented and serious public health situation [11,12]. In addition to arboviruses with established circulation in urban environments, MAYV (predominantly circulating in Amazonia) has been extensively studied, and its potential for urban establishment raises public health concerns [4,13].

In North America, the major circulating arbovirus is West Nile virus (WNV), which has been reported to cause disease in humans and horses in the United States and Canada for over two decades [14,15]. Besides North America, WNV has also been reported in Europe, Africa, and South America [15]. In Brazil, WNV circulation is not fully understood, but it has been reported in nearly all parts of the country, with 110 human cases over the past 10 years, detection in birds, and a fatal case in a horse. [16].

Given the recent ZIKV epidemic in Brazil and the ongoing circulation of MAYV and WNV, even if limited, it is essential to deepen our understanding of infection dynamics and mechanisms of coinfections and superinfections. These viruses have significant epidemic potential and can co-circulate across Latin America [4,13]. *In vitro* coinfection of DENV, CHIKV, MAYV, ZIKV, WNV, and Usutu virus (USUV) has been demonstrated in Vero, C6/36, GN-R, and Huh7 cells [6,17–19], and a few human cases of double and triple infections have been reported [20,21]. The effects of coinfection on the epidemiology, pathogenesis, and evolution of these viruses remain unclear, as challenges such as diagnosis difficulties, symptom overlap, and limited clinical data hinder understanding of the scope and impact of these events [6,22].

The widespread circulation of various arboviruses combined with large vector populations increases the likelihood of coinfection (when a vector or human is infected by more than one virus at the same time) and superinfection (when a vector or human is infected by more than one virus sequentially) [23,24]. The mechanisms behind these processes are not fully understood; however, four different scenarios are proposed for cells, mosquitoes, and humans: i) increased replication of both viruses (i.e., enhancement), ii) decreased replication of both viruses (i.e., inhibition), iii) competition between viruses (i.e., interference), and iv) no effect on replication (i.e., neutral) [22,23]. This means that when viruses compete for resources, one might be seen as dominant because it interferes with the replication of the other virus, while continuing to replicate as if it were a single infection. To shed light on the coinfection dynamics of highly relevant arboviruses worldwide, this study investigated single infection, coinfection, and superinfection involving ZIKV and WNV, and ZIKV and MAYV, in mammalian cells. And this highlights the importance of studying basic *in vitro* replication interactions in combination with WNV and neotropical arboviruses such as ZIKV and MAYV.

## Materials and methods

### Cells and viral strain

The experiments were performed using viral stocks of ZIKV (PRVABC59, GenBank acc. KU501215), WNV (NY99, GenBank acc. AF196835), and MAYV (TRVL 15537, GenBank acc. KP842810). Viral stocks were grown in Vero CCL81 cells, kidney epithelial cells from *Cercopithecus aethiops* (BEI Resources), under Containment Level 3 (CL3) conditions at Brock University. Cells were cultivated in culture flasks and, at 80–90% confluency, were infected with the specific virus (MAYV, WNV, or ZIKV) at a multiplicity of infection (MOI) of 0.02. The Vero cell passages (P) used ranged from P15 to P30. To evaluate the quality of the cells employed, they were routinely tested for mycoplasma contamination using the Mycoplasma Pro PCR Detection Kit (Applied Biological Materials, Inc.). Infected cells were maintained in Dulbecco’s Modified Eagle’s Medium (DMEM) high glucose (Gibco), supplemented with 2% FBS (Gibco), and 2% glutamine-penicillin-streptomycin solution (Gibco), and were incubated at 37 °C + 5% CO₂ conditions. Following the observation of a notable cytopathic effect (CPE), the culture media from infected cells were centrifuged at 5,000 g for 30 minutes at 4 °C. The clarified stock was aliquoted and kept at −80 °C until further use.

### Viral titration by Plaque assay

Plaque assay was performed as described in Agbulos et al. (2016) [25], after three days of incubation at 37 °C with 5% CO_2_ for WNV and MAYV, and five days for ZIKV, the media was removed. The cell monolayers were stained with a solution containing 1% crystal violet (Sigma), 20% formaldehyde (Sigma), and 30% ethanol (Sigma) in Phosphate-buffered saline (PBS) (Sigma) for 30 minutes to fix and facilitate plaque visualization and counting. Stock viral titers were calculated, resulting in 7.5 × 10^6^ plaque-forming units per milliliter (PFU/mL) for ZIKV, 4.0 × 10^7^ PFU/mL for WNV, and 2.0 × 10^7^ PFU/mL for MAYV. ZIKV, WNV, and MAYV viral stocks were propagated in passage P4. For all experiments, stock viral titers were used in calculations to ensure that cells were inoculated at equivalent multiplicities of infection (MOI).

### *In vitro* single infections

For single infection experiments, Vero cells were seeded and, upon reaching 80–90% confluence (24–48 hours), were inoculated with one of ZIKV, WNV, or MAYV at an MOI of 0.01. Cells were placed in the incubator for one hour and gently rocked every 15 minutes. Following the viral adsorption period, cells were washed with PBS and maintained in DMEM supplemented with 2% FBS. Cells were cultured for six days following infection with no media change. In addition to the conditions of interest, a negative control was included in all experiments. Cells were monitored daily, and images were captured to visualize CPE.

### *In vitro* simultaneous coinfections (CI) and superinfections (SI)

In coinfection (CI) experiments, both viruses – ZIKV and WNV, or ZIKV and MAYV – were allowed access to cells simultaneously during infection, following the protocol described in the previous section. Under superinfection (SI) conditions, one virus was presented first, followed by the other after one day. The final MOI of 0.01 was used for each virus in all pairings, and both orders of superinfection were investigated: i) SI ZIKV/WNV, where ZIKV was presented first, followed by WNV; ii) SI WNV/ZIKV, where WNV was presented first, followed by ZIKV; iii) SI ZIKV/MAYV, where ZIKV was presented first, followed by MAYV; and iv) SI MAYV/ZIKV, where MAYV was presented first, followed by ZIKV. Single infection conditions were also included for all three viruses to assess the effects of coinfections. In addition to the conditions of interest, a negative control was added to all experiments. Cells were monitored daily, and images were captured to visualize CPE.

### Image capture and cell-covered areas calculation

Observation of CPE during Vero cell infections was observed in three randomly selected areas of each flask, and images were taken daily for six days after viral infection. Images were obtained using a Motic AE 2000 inverted microscope (Motic) and a Google Pixel 3 smartphone camera. Images were analyzed using open-source Fiji software [26] and the Maximum Entropy thresholding method [27]. A morphological dilation process was applied to binary images to better delineate and visualize the area covered by cells. Next, the percentage of white and black pixels was calculated to represent the proportion of cells to background in the image, respectively, to estimate the amount of cell death (cell detachment).

### RNA Isolation and RT-qPCR

RNA extraction was performed using the Total RNA Purification Kit (Norgen Biotek) according to the manufacturer’s instructions. RNA was extracted from 100 μL of each sample and eluted in 100 μL of nuclease-free water. RNA samples were kept at −80 °C until RT-qPCR was performed.

Each RT-qPCR reaction was carried out using the QuantiNOVA Probe RT-PCR Kit (Qiagen) using primers and probes, as described previously for ZIKV [26], WNV [27], and MAYV [28] (Table 1). The reactions were carried out in a final volume of 10 μL using a CFX96 Real-time PCR system (Bio-Rad) under the following conditions: 50 °C for 10 min, followed by 95 °C for 10 min, and then 40 cycles of 95 °C for 10 s and 60 °C for 30 s. Samples were tested in duplicate, with an automatic threshold and baseline. Results were analyzed using CFX Maestro Software, and samples with cycle quantification (Cq) values  ≤ 37 were considered positive. The quality parameters of the runs were monitored in accordance with the MIQE Guide [32].

**Table 1:** Description of primers and probes used in RT-qPCR for Zika, West Nile, and Mayaro virus detection.

Virus	Target	Primer	Sequence	Final Conc.
ZIKV	prM protein	ZIKV – F	TTGGTCATGATACTGCTGATTGC	650 nM
		ZIKV- R	CCTTCCACAAAGTCCCTATTGC	650 nM
		ZIKV – P	(FAM)CGGCATACAGCATCAGGTGCATAGGAG	250 nM
WNV	3’UTR	WNV – F	CAGACCACGCTACGGCG	1000 nM
		WNV – R	CTAGGGCCGCGTGGG	1000 nM
		WNV – P	(FAM)TCTGCGGAGAGTGCAGTCTGCGAT	125 nM
MAYV	nsp1 protein	MAYV – F	CACGGACMTTTTGCCTTCA	500 nM
		MAYV – R	AGACTGCCACCTCTGCTKGAG	500 nM
		MAYV – P	(HEX)ACAGATCAGACATGCAGG	200 nM

**Note:** ZIKV- Zika virus, WNV-West Nile virus, MAYV- Mayaro virus, prM- Precursor membrane protein, 3’UTR-3’ Untranslated Region, nsp1- Non-structural protein 1, F-Forward, R-Reverse, nM-nanomolar. Primers and probes are taken from Lanciotti et al. 2008 [29] for ZIKV, Lanciotti et al. 2000 [30] for WNV, and Naveca et al. 2017 [31] for MAYV.

### Data analysis

Viral RNA quantification was calculated by generating a standard curve with previously titrated samples. The trendline equation obtained from the standard curve was used to calculate the Log Starting Quantity (LSQ) for all samples, based on Cq values. Therefore, LSQ values refer to virus titer (i.e., LSQ of 6 refers to 10^6^ PFU/mL). To understand the actual impact of concurrent infections on RNA production, mean LSQ values found for experimental conditions (coinfections or superinfections) were divided by the corresponding LSQ values for single infections to generate a relative LSQ value. This ratio indicates whether RNA production is increased (> 1) or decreased (< 1), compared to the baseline of single infections. To compare two groups, a Welch’s t-test was used, and when more than two conditions were compared, a one-way ANOVA was used. Results were considered significant when the p-value < 0.05. All statistical tests and graphics were performed using GraphPad Prism 10.0.2 software. (GraphPad Software).

## Results

### Single infections

**Fig 1:**
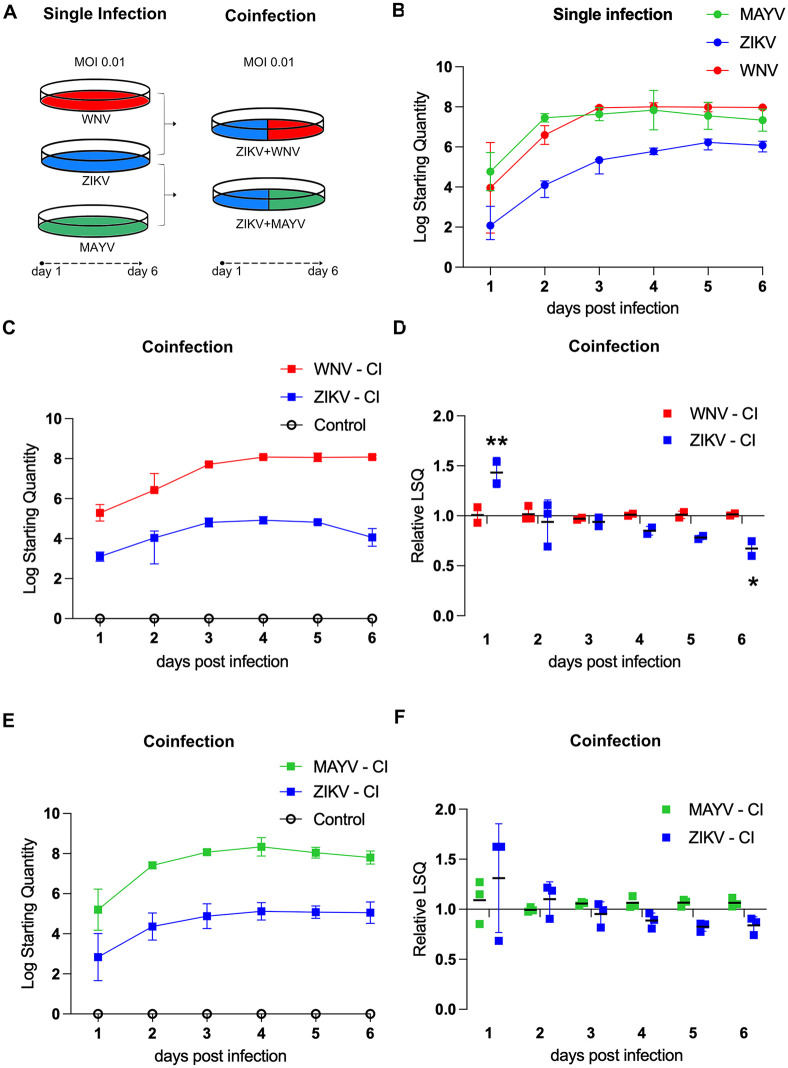
Experimental design and Log Starting Quantity (LSQ) data visualization of the results from single infection and coinfection of Zika, West Nile, and Mayaro viruses in Vero cells. **Note:** (A) Experimental design of single infection and coinfection assays of Zika (ZIKV), West Nile (WNV), and Mayaro (MAYV) viruses; (B) Graph of replication curves for ZIKV, WNV, and MAYV over 6 days post-single infection in Vero cells, shown in Log Starting Quantity (LSQ) values; (C) Graph of replication curves for ZIKV and WNV coinfection over 6 days post-infection in Vero cells, shown in LSQ values. (D) Graph of relative LSQ for ZIKV and WNV coinfection compared with single infection; (E) Graph of replication curves for ZIKV and MAYV coinfection over 6 days post-infection in Vero cells, shown in LSQ values. (F) Graph of relative LSQ for ZIKV and MAYV coinfection compared with single infection. MOI – Multiplicity of infection; CI -Coinfection, Control- Vero cells with no virus. The graphs in B, C, D, E and F represent the mean  ±  S.D. of three independent experiments for ZIKV and MAYV and two for WNV (three technical replicates were performed in each experiment). Statistical analysis was performed using Graph Pad Prism 10 (* *p < 0.05*, ** *p < 0.01*, *** *p < 0.001*, **** *p < 0.0001*).

The first step was to examine the replication patterns of ZIKV, WNV and MAYV during single infections in Vero cells (Fig 1A). Over six days, ZIKV LSQ values ranged from 2.16 to 6.16, peaking at 5 days post-infection (dpi). WNV LSQ values ranged from 3.96 to 8.00, with the highest at 4 dpi. Similarly, MAYV LSQ values ranged from 4.77 to 7.84, again peaking at 4 dpi (Fig 1B). At 1 dpi, ZIKV LSQ levels were significantly lower than those of MAYV, and this difference remained through 4 dpi. The p-values were *p = 0.0039, p < 0.0001, p = 0.006,* and *p = 0.0435* for days 1, 2, 3, and 4, respectively. ZIKV LSQ values were also lower than those of WNV, but the difference was only statistically significant at 2 and 3 dpi with *p = 0.0033* and *p = 0.0055,* respectively. No significant differences were observed between the replication patterns of WNV and MAYV.

**Fig 2:**
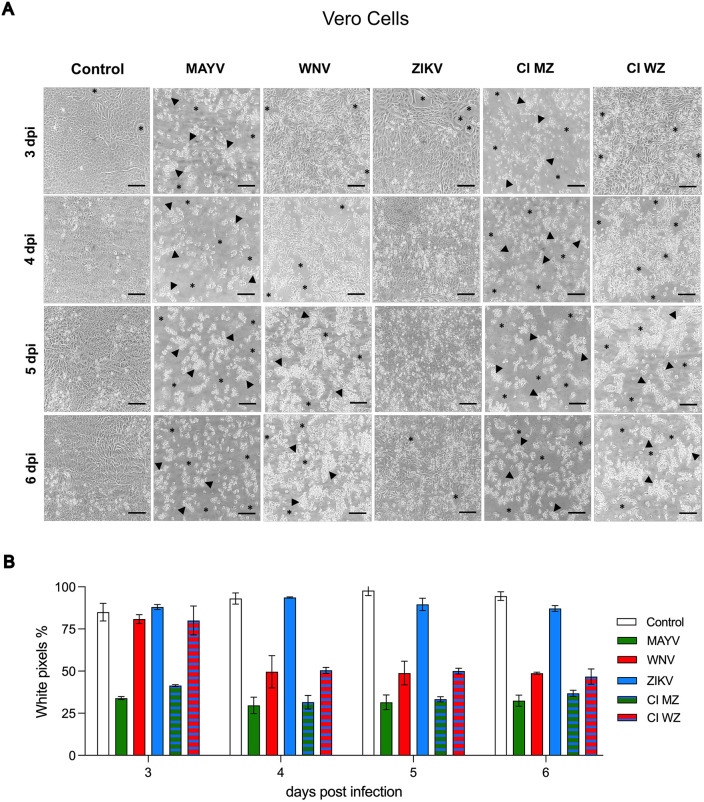
Cytopathic effect and quantification of Vero cell monolayer integrity during single and coinfections with Zika, West Nile, and Mayaro viruses. **Note:** (A) Panel of images showing cytopathic effect in Vero cells infected with Zika, West Nile, and Mayaro viruses in single and coinfection. (B) Graphical representation of Vero cell-covered areas during single and coinfections with Zika, West Nile, and Mayaro viruses; MAYV- Mayaro virus, WNV-West Nile virus, ZIKV- Zika virus, CI MZ- Coinfection between Zika and Mayaro, CI WZ- Coinfection between Zika and West Nile, dpi- day post-infection, Control- Vero cells with no virus, %-percentage. Black arrows indicate CPE, and black stars indicate empty spaces. Scale bar: 100µm.

During CPE monitoring, ZIKV was the last virus to exhibit CPE, with increased cell death observed from 4 dpi onward, along with stressed, stretched attached cells. However, the day with the highest CPE was 6 dpi, although the cellular monolayer was still present. For WNV, CPE from 3 dpi onwards was strong, and the cellular monolayer was almost absent at 6 dpi. Finally, MAYV proved to be the most virulent virus for Vero cells, with significant cell death observed at 3 dpi, resulting in almost complete detachment (Fig 2A).

### Coinfections

Vero cells coinfected with ZIKV and WNV produced higher levels of WNV RNA than ZIKV RNA on every day studied. A statistically significant difference in mean LSQ was found between ZIKV and WNV on all days (*p = 0.0127, p < 0.0001, p < 0.001, p = 0.0004, p = 0.0004, p < 0.001*). LSQ values for ZIKV ranged from 3.1 to 4.92, with the replication peak occurring at 4 dpi (Fig 1C). For WNV, the LSQ range was 5.29 to 8.08, with replication peaks on 4 dpi and 6 dpi (Fig 1C). ZIKV RNA production in Vero cells coinfected with WNV did not reach the levels obtained in single infections (Fig 1C vs Fig 1B).

To assess the actual impact of coinfection on viral RNA levels, relative LSQ values were calculated. The comparison revealed a significant difference in ZIKV RNA production between Vero cells co-infected with ZIKV and WNV and those infected with only one virus. Although ZIKV and WNV coinfection initially showed a significant 43.3% increase in ZIKV RNA levels compared to single infections (*p < 0.0001*), ZIKV RNA production declined as the coinfection progressed (Fig 1C). A significant decrease was only observed on the last day examined (*p = 0.0049*); however, ZIKV RNA levels started to decline at 4 dpi, with approximately a 15% decrease at this time point. Levels continued to drop at 5 dpi, with a mean reduction of 21.7% and finally fell below the statistically significant threshold with a 32.7% decrease by 6 dpi. WNV RNA levels remained unaffected during coinfection with ZIKV (Fig 1D).

The CPE observed in the coinfection with ZIKV and WNV was more evident from 3 to 6 dpi compared to the single ZIKV infection. Compared to the single WNV infection, the cells presented the characteristics of stress, death, and morphological alterations. The evaluation of the percentage of white pixels showed that from 4 dpi onwards, the number of attached cells in the ZIKV and WNV coinfection was similar to that in the single WNV infection. Meanwhile, cells in the ZIKV single infection maintained an attached cellular monolayer for a longer period (Fig 2).

In coinfected Vero cells with ZIKV and MAYV, MAYV RNA concentration was higher than ZIKV RNA levels on every day (with p-values ranging from *p = 0.0041* to *p < 0.0001)*. Viral RNA production increases as the coinfection progresses, and it is highest at 4 dpi for both viruses, with an LSQ range of 2.83 to 5.12 for ZIKV and 5.20 to 8.33 for MAYV (Fig 1E). Compared to single infections, this is an earlier peak for ZIKV RNA production.

Regarding the Relative LSQ, no statistically significant difference was observed in viral RNA production between Vero cells coinfected with ZIKV and MAYV and those infected with only one virus. However, a 17.4% decrease in ZIKV RNA levels was noted at 5 dpi when compared to single infection (Fig 1F).

The CPE observed in the ZIKV and MAYV coinfection was more evident on all days when compared to the single ZIKV infection. Compared to the single MAYV infection, the cells exhibited the same CPE characteristics, including cell death and morphological alterations. The evaluation of the percentage of white pixels showed that on all days, the number of adhered cells in the ZIKV and MAYV coinfection was lower than in single infections (ZIKV, MAYV, and WNV) and compared with the ZIKV and WNV coinfection (Fig 2).

### Superinfections

**Fig 3:**
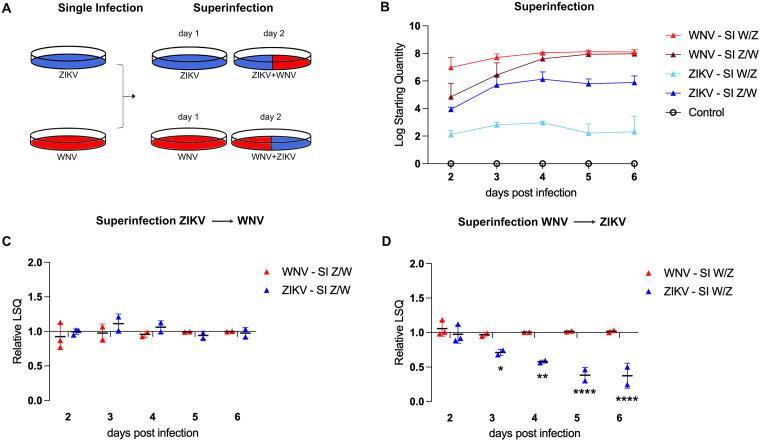
Experimental design and Log Starting Quantity (LSQ) data visualization of results from superinfection with Zika and West Nile in Vero cells, in both orders of infection. **Note:** (A) Experimental design of superinfection assays for Zika (ZIKV) and West Nile (WNV); (B) Graph of replication curves for ZIKV and WNV during superinfection over 6 days post-infection in Vero cells, shown as Log Starting Quantity (LSQ) values; (C) Graph of relative LSQ for ZIKV and WNV superinfection compared with single infection, with ZIKV inoculated first; (D) Graph of relative LSQ for ZIKV and WNV superinfection compared with single infection, with WNV inoculated first; SI-Superinfection, Z-Zika virus, W-West Nile virus, Control- Vero cells with no virus, LSQ-Log Starting quantity. The graphs in B, C and D show the mean  ±  S.D. from three independent experiments on days 1 and 2, and from two experiments on days 3, 4, 5 and 6 (three technical replicates were performed in each experiment). Statistical analysis was performed using Graph Pad Prism 10 (* *p < 0.05*, ** *p < 0.01*, *** *p < 0.001*, **** *p < 0.0001*).

To investigate the impact of superinfection on viral RNA production, cells were infected by one of the viruses in the pairing, followed by the other one day later (Fig 3A). Both orders of virus exposure were investigated for ZIKV and WNV, as well as for ZIKV and MAYV superinfections.

During the superinfection with ZIKV and WNV, ZIKV RNA production was influenced by the order of virus exposure. ZIKV LSQ was most affected in SI W/Z, peaking at 4 dpi with LSQ levels ranging from 2.11 to 2.97 (Fig 3B). When ZIKV was the first to infect the cells (SI Z/W), LSQ ranged from 3.94 to 6.12, with the peak replication also at 4 dpi. In SI W/Z conditions, we observed a statistically significant decrease in ZIKV RNA levels (*p < 0.0001)* in Vero cells as early as 3 dpi. ZIKV RNA levels continued to decline as superinfection progressed, with average decreases of 42.1% at 4 dpi and 61.8% at 5 dpi. The largest reduction occurred at 6 dpi, when ZIKV RNA levels in Vero cells under the SI W/Z condition were 62.5% lower than those in single ZIKV infections. The suppression of ZIKV replication in the presence of WNV was confirmed by comparing the relative LSQ values, which showed that from 3 dpi onwards, ZIKV RNA production was significantly lower compared to a single infection (*p = 0.0214, p = 0.0010, p < 0.0001, p < 0.0001*). In superinfection conditions where ZIKV was the first to infect Vero cells (SI Z/W), RNA production dynamics were not significantly different from those seen in single-infected cells (Fig 3C).

WNV replication dynamics in Vero cells were not affected by the presence of ZIKV, and RNA levels peaked after WNV had been in the cells for 5 days in both SI conditions. This translates into the highest WNV RNA production at 6 dpi and 5 dpi for Z/W and W/Z, respectively, as shown in Fig 3B. In the SI W/Z experiment, LSQ values for WNV ranged from 6.98 to 8.12 and in SI Z/W, from 4.85 to 7.97 (Fig 3B). Relative to single infections, in both SI Z/W and SI W/Z scenarios, WNV production was unchanged in both SI Z/W (Fig 3C) and SI W/Z (Fig 3D) scenarios.

**Fig 4:**
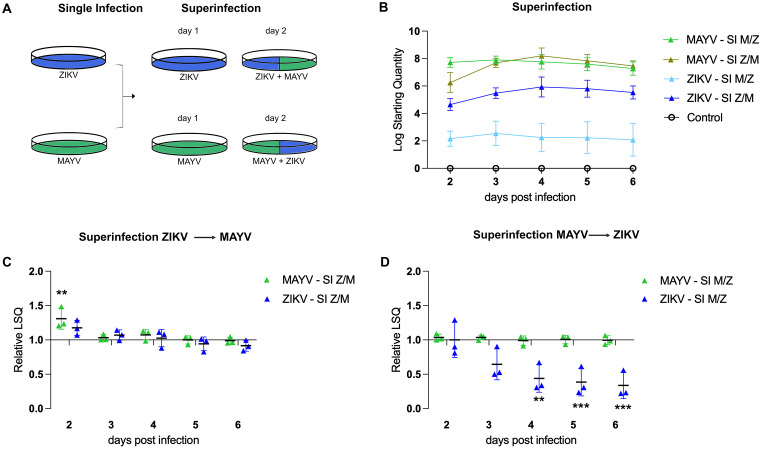
Experimental design and Log Starting Quantity (LSQ) data visualization of the results from superinfection of Zika and Mayaro in Vero cells in both orders of infection. **Note:** (A) Experimental design of superinfection assays for Zika (ZIKV) and Mayaro (MAYV); (B) Graph of replication curves for ZIKV and MAYV during superinfection over 6 days post-infection in Vero cells, shown as Log Starting Quantity (LSQ) values; (C) Graph of relative LSQ for ZIKV and MAYV superinfection compared with single infection, with ZIKV inoculated first; (D) Graph of relative LSQ for ZIKV and MAYV superinfection compared with single infection, with MAYV inoculated first; SI-Superinfection, Z-Zika virus, W-West Nile virus, Control- Vero cells with no virus, LSQ-Log Starting quantity. The graphs in B, C and D show the mean  ±  S.D. from three independent experiments (three technical replicates were performed in each experiment). Statistical analysis was performed using Graph Pad Prism 10 (* *p < 0.05*, ** *p < 0.01*, *** *p < 0.001*, **** *p < 0.0001*).

Superinfection experiments with ZIKV and MAYV (Fig 4A) revealed that ZIKV RNA production also depends on the order of virus exposure. ZIKV LSQ was most impacted in SI M/Z conditions, with LSQ levels ranging from 2.16 to 2.55. When ZIKV was the initial virus infecting the cells (SI Z/M), LSQ ranged from 4.65 to 5.96. The peak of ZIKV RNA production occurred at 4 dpi in both superinfection scenarios (Fig 4B). In SI M/Z conditions, we observed a statistically significant decrease in ZIKV RNA levels (*p <* *0.0001*) in Vero cells as early as 3 dpi, similar to the reduction seen in SI W/Z conditions. The most notable difference appeared at 6 dpi, when Vero cells in the SI M/Z condition produced 41.39% less ZIKV RNA than single-infected cells. The suppression of ZIKV replication by MAYV was confirmed by comparing relative LSQ values, which showed that from 4 dpi onward, ZIKV RNA production was significantly lower than in single infections (*p = 0.0013, p = 0.0005, p = 0.0002*) (Fig 4D). In superinfection conditions where ZIKV was the first to infect Vero cells (SI Z/M), the RNA production dynamics did not differ significantly from those in single-infected cells (Fig 4C).

MAYV replication dynamics in Vero cells were not affected by the presence of ZIKV, and RNA levels peaked after MAYV had been in cells for 3 days (i.e., at 4 dpi in SI Z/M and 3dpi in SI M/Z conditions). In SI M/Z, LSQ ranged from 7.27 to 7.9. During SI Z/M conditions, the LSQ range was from 6.24 to 8.19 (Fig 4B). Relative LSQ analysis indicated that, compared to single infections, MAYV RNA production was significantly elevated at 2 dpi during SI Z/M (*p = 0.0026*), but showed no further change beyond 3 dpi (Fig 4C).

The CPE observed in all superinfection scenarios was more prominent on all days compared to the single infections from 3 dpi to 6 dpi. Morphological changes, stress, and cell death were noted. Images of CPE during superinfections were not captured because the cell monolayer was entirely altered, making focusing and image capture difficult.

## Discussion

The global spread of mosquito-borne viruses, especially in tropical and sub-tropical regions, has been characterized by their frequent emergence and reemergence, along with rapid expansion across regions [33]. This pattern has led to the simultaneous presence of multiple arboviral pathogens in certain areas, which can cause coinfections and superinfections in both vectors and humans [22]. This study focuses on key clinical arboviruses: ZIKV, WNV, and MAYV. The confirmed presence of ZIKV in Latin America, especially in Brazil, the potential emergence of MAYV in urban areas, and the uncertain circulation of WNV over the past decade emphasize the need for *in vitro* coinfection studies as a preliminary step to understand viral replication during multiple exposures. Our findings suggest that ZIKV RNA levels may be reduced during coinfection with WNV and MAYV under the experimental conditions evaluated. Overall, ZIKV has the lowest replication rate, while WNV shows the highest.

In single infections and coinfection assays, ZIKV replication was lower compared to WNV and MAYV. This same pattern was observed by Rückert et al. [6], who described the replication kinetics of ZIKV, CHIKV, and DENV in Vero cells under single infection conditions with a 10 times higher MOI (0.1) than the one used here and found similar viral titers for ZIKV. Brustolin et al. [17] investigated for the first time the CI and SI scenarios with the ZIKV and MAYV combination. They observed that ZIKV replication aligns with the results found here and noted that in the CI and SI M/Z scenarios, ZIKV experienced severe interference and exclusion, even when testing different intervals for sequential infection (two and 12 hours). Interestingly, in this same study, the same pattern was observed using C6/36 insect cells (*Aedes albopictus* cells). However, in Aag2 cells *(Aedes aegypti* cells), neither ZIKV nor MAYV experienced interference. A study conducted with ZIKV and DENV found that during sequential infection with these viruses, ZIKV experienced a decrease in viral titer compared to single infection in C6/36 cells from 2 dpi to 6 dpi [34]. The study in question performed the SI D/Z with a 12-hour interval (we used a 24-hour interval here) and observed results similar to those of our SI W/Z experiment. Another important arbovirus, CHIKV, circulates in tropical regions of the globe and belongs to the same family and genus as MAYV (*Alphavirus* from the *Togaviridae* family). Göertz et al. [35] studied the coinfection of ZIKV and CHIKV in Vero, C6/36, and Aeg2 cells. The results corroborated our findings, indicating that in the presence of an *Alphavirus*, ZIKV replicates at lower levels. However, differences in cytopathic effects and monolayer integrity between single- and coinfection conditions may have influenced the viral RNA levels detected during some experiments. Therefore, reductions in viral RNA levels cannot be interpreted exclusively as evidence of direct viral interference or classical superinfection exclusion mechanisms.

Here, we describe the co-infection of ZIKV with WNV and highlight the importance of studying this combination. The interaction between *Orthoflavivirus* has been reported, and as WNV has been circulating in South America for years [16], the possibility of coinfection with a virus like ZIKV cannot be discounted. Under the experimental conditions evaluated here, WNV RNA levels were not markedly affected by the presence of ZIKV. Svyatchenko et al. [19] have reported ZIKV and WNV coinfection in Vero E6 cells; however, the effects of sequential and simultaneous infections with these arboviruses remain poorly understood. Some studies have already explored co-infection of WNV with Usutu virus (USUV) and found that *in vitro* co-infection experiments show WNV has higher viral fitness in mammalian, avian, and mosquito cells compared to USUV [18,36]. Furthermore, Gallichotte et al. [37] studied Saint Louis encephalitis virus (SLEV) and WNV and found that in Vero cells, coinfection has minimal impact on WNV RNA levels at either a low (0.01) or high (1) MOI; however, at a high MOI, WNV significantly decreases SLEV RNA. Other studies have explored the co-infection of WNV with Bamaga virus [38] and Culex Flavivirus, an insect-specific virus (ISV) [39]. However, these studies showed that interference with WNV replication occurred only when the viruses were present at substantially higher concentrations than WNV in C6/36 cells.

Several studies on coinfection and superinfection using mosquito experiments investigate how multiple exposures affect vector competence by evaluating infection, dissemination, and transmission rates. These studies often require *in vitro* research to clarify interactions in viral replication. Brustolin et al. [17] and Rückert et al. [6] showed that ZIKV replication was reduced when one of MAYV, DENV, or CHIKV was present (either CI or SI) in *Aedes aegypti* mosquitoes. Wang et al. [36] found that USUV replication in *Culex pipiens* mosquitoes was influenced by the presence of WNV, both in CI and SI conditions. During SI vector experiments, the second virus showed reduced fitness. Similarly, a single infection vs. CI study of WNV and SLEV in *Culex* *quinquefasciatus* and *Culex tarsalis* mosquitoes indicated that simultaneous infection does not affect the infection rates in mosquitoes [37]. Taking all these findings into account, it is important to note that the dynamics of arbovirus replication in mosquitoes are complex and can be influenced by multiple factors, including mosquito species, viral strain, temperature conditions during infection, microbiota composition, initial viral load, and the interval between exposures [28,33,40]. The effects are highly context-dependent and most evident at low MOI. This variability among different systems may explain some of the ambiguity in the literature regarding the impact of coinfection. We emphasize that *in vitro* systems do not fully replicate the complexity of virus-host and vector-virus interactions observed *in vivo*. In addition, the exclusive use of a mammalian cell line is a limitation of this study, and future investigations using mosquito-derived cell lines will be important for better understanding how these interactions may occur in vectors and hosts. However, they can be helpful as an initial step toward understanding viral replication in various contexts, such as coinfections and superinfections.

The impact of coinfection on different aspects of virus biology, host immune response, and transmission by mosquitoes is not yet fully understood. Although there are many potential viral effects of coinfection, they can generally be grouped into four categories: i) increased replication of both viruses, ii) decreased replication of both viruses, iii) competition between viruses, and iv) no effect on replication [20,35,36]. These outcomes are shaped not only by the viral replication strategies and immune evasion mechanisms, but also by host-specific factors and the ability of intracellular machinery and innate immune responses to detect and distinguish single infections from coinfections [22,41]. Because of these and other factors, coinfection may alter infection dynamics within cells, vectors, and hosts. Gaining a clear understanding of these mechanisms is crucial for studying outcomes during both simultaneous and sequential infections. A better understanding of these mechanisms may help clarify the potential implications of coinfection for co-transmission by mosquitoes, host infection dynamics, disease progression, and viral evolution.
